# Adaptive optimal basis set for BCG artifact removal in simultaneous EEG-fMRI

**DOI:** 10.1038/s41598-018-27187-6

**Published:** 2018-06-11

**Authors:** Marco Marino, Quanying Liu, Vlastimil Koudelka, Camillo Porcaro, Jaroslav Hlinka, Nicole Wenderoth, Dante Mantini

**Affiliations:** 1 Research Center for Motor Control and Neuroplasticity, KU Leuven, Leuven, Belgium; 2 Department of Experimental Psychology, University of Oxford, Oxford, United Kingdom; 3 Neural Control of Movement Laboratory, ETH Zurich, Zurich, Switzerland; 4 National Institute of Mental Health, Klecany, Czech Republic; 5 LET’S-ISTC, National Research Council, Rome, Italy; 6 Birmingham University Imaging Centre (BUIC), School of Psychology, University of Birmingham, Edgbaston, Birmingham United Kingdom; 7 Institute of Computer Science, Academy of Sciences of the Czech Republic, Prague, Czech Republic; 8 Functional Neuroimaging Laboratory, IRCCS San Camillo Hospital Foundation, Venice Lido, Italy

## Abstract

Electroencephalography (EEG) signals recorded during simultaneous functional magnetic resonance imaging (fMRI) are contaminated by strong artifacts. Among these, the ballistocardiographic (BCG) artifact is the most challenging, due to its complex spatio-temporal dynamics associated with ongoing cardiac activity. The presence of BCG residuals in EEG data may hide true, or generate spurious correlations between EEG and fMRI time-courses. Here, we propose an adaptive Optimal Basis Set (aOBS) method for BCG artifact removal. Our method is adaptive, as it can estimate the delay between cardiac activity and BCG occurrence on a beat-to-beat basis. The effective creation of an optimal basis set by principal component analysis (PCA) is therefore ensured by a more accurate alignment of BCG occurrences. Furthermore, aOBS can automatically estimate which components produced by PCA are likely to be BCG artifact-related and therefore need to be removed. The aOBS performance was evaluated on high-density EEG data acquired with simultaneous fMRI in healthy subjects during visual stimulation. As aOBS enables effective reduction of BCG residuals while preserving brain signals, we suggest it may find wide application in simultaneous EEG-fMRI studies.

## Introduction

Multimodal integration of simultaneously collected electroencephalography (EEG) and functional magnetic resonance imaging (fMRI) data represents a powerful approach for shedding light on human brain function^1–4^. The strength of EEG-fMRI integration relies on the possibility of assessing brain dynamics at different spatial-temporal scales^2,5,6^. Indeed, EEG records rapid changes in potentials over the scalp with high temporal resolution^7^, and fMRI measures slow hemodynamic variations underlying neural activity with high spatial accuracy and resolution^8,9^. Typical examples demonstrating the potential advantages of simultaneous EEG-fMRI are the mapping of neural oscillations during task^10,11^, rest^12–14^, and sleep^15,16^, as well as the accurate localization of epileptic events^17–19^.

Despite the clear advantages of EEG-fMRI integration, the combination of the two techniques also raises important technical challenges^14,20–22^. More specifically, EEG signals recorded during simultaneous fMRI scanning are contaminated by strong artifacts^23–26^, which complicate the characterization of the measured brain signals and should therefore be removed before continuing with any further analysis. The two main artifacts mixed in the EEG recordings are the gradient artifact^27,28^, which is generated by the image acquisition process, and the ballistocardiographic (BCG) artifact^29–31^, produced by micro-movements of the scalp due to blood pulsation.

Gradient-related artifact events can be removed in a relatively easy manner, as they are highly reproducible. The average artifact subtraction (AAS) algorithm^27^, calculated by averaging the signal from several scanning periods in each EEG channel, usually yields acceptable artifact reduction. The attenuation of the BCG artifact, on the other side, still represents an open issue due to its complex spatio-temporal properties^32,33^. Several concurrent mechanisms contribute to the BCG artifact^33^, such as pulse driven expansion of the scalp^30,33,34^, cardiac pulse driven rotation of the head inside the strong static magnetic field^33–35^ and the Hall effect of the pulsatile blood flow^31^ that, as an electrically conductive fluid, induces changes of the measured voltage on the scalp surface.

As BCG artifact occurrences are approximately time-locked to cardiac activity, AAS can be applied by averaging the EEG data epoched across successive cardiac cycles^29^. However, this method cannot take into account the large variability of the BCG artifact shape in any single EEG channel. To account for this problem, an optimal basis set (OBS) approach was proposed as an extension of AAS^36^. Specifically, principal component analysis (PCA) is applied to EEG segments time-locked to the cardiac events, and the first components are used for adaptive artifact removal^36^. An open issue in the use of OBS is the selection of the number of principal components (PCs) to be removed from the EEG data. The first 3 or 4 components are typically removed when using OBS, but there is no consensus on whether this can yield satisfactory results for all channels of the same dataset and for all datasets^37^. Also, the performance of OBS is hampered by the fact that events for data epoching are typically identified on a simultaneously recorded ECG signal, assuming a fixed delay between the cardiac event and the artifactual occurrence in the EEG recording. The variability between ECG and BCG events^38–40^ may therefore have negative impact on the OBS performance.

Independent component analysis (ICA), a signal processing technique that can recover independent components (ICs) that are linearly mixed in a set of simultaneously recorded signals^41^, has also been proposed for the removal of the BCG artifact in multi-channel EEG data collected during fMRI scanning^32,42^. Following ICA, the components associated with BCG activity are either manually or automatically identified, and then subtracted with appropriate weights from the EEG data^42–44^. A first challenge in the use of ICA is the definition of unanimous criteria for the selection of the artifactual components^37^. Furthermore, ICA-based artifact removal may be sub-optimal when some ICs still contain a mixture of neural activity and artifacts^43,45^. This may happen when dealing with BCG artifacts, which are characterized by varying spatial topography across time^30,32,38^. In this case, the basic ICA assumption of instantaneous mixture of ICs is clearly not fulfilled, as the BCG events are not perfectly aligned in time. This problem is present in any EEG dataset collected during fMRI scanning, but it can be particularly appreciated when using high-density EEG montages, which have large scalp coverage^38^.

Considering the intrinsic limitations of AAS, OBS and ICA, an approach that has been suggested to improve BCG artifact removal is the combination of AAS/OBS with ICA^32,43,46^. AAS/OBS can be applied either before^32^ or after ICA decomposition^43,46^. In the former case, ICA is run on EEG recordings in which the BCG contribution has been already attenuated; if residual BCG components are detected, they are removed from the data^32^. The detection of these residual components is however a challenging task, as the typical shape of the BCG is not preserved following AAS/OBS. An incorrect classification may lead in some cases to undesirable signal loss and distortion. A second possibility consists of performing an EEG data decomposition by ICA, an attenuation of the BCG contribution in each component by AAS/OBS, and a weighted recombination of the components to generate BCG-cleaned recordings^43^. In this case, the accuracy of the ICA decomposition may be affected by the spatial non-stationarity of the BCG sources. Although the use of either AAS or OBS on each component may represent an effective approach to BCG artifact attenuation, it can also lead to substantial signal distortions if neural activity is not completely uncorrelated from the BCG signal. Based on the considerations above, we suggest that using different methods on the same dataset can certainly lead to stronger artifact attenuation, but the distortion of true brain activity will possibly be larger. As such, BCG artifact removal methods that can achieve substantial artifact attenuation while preserving brain activity are still warranted^43^.

**Figure 1:**
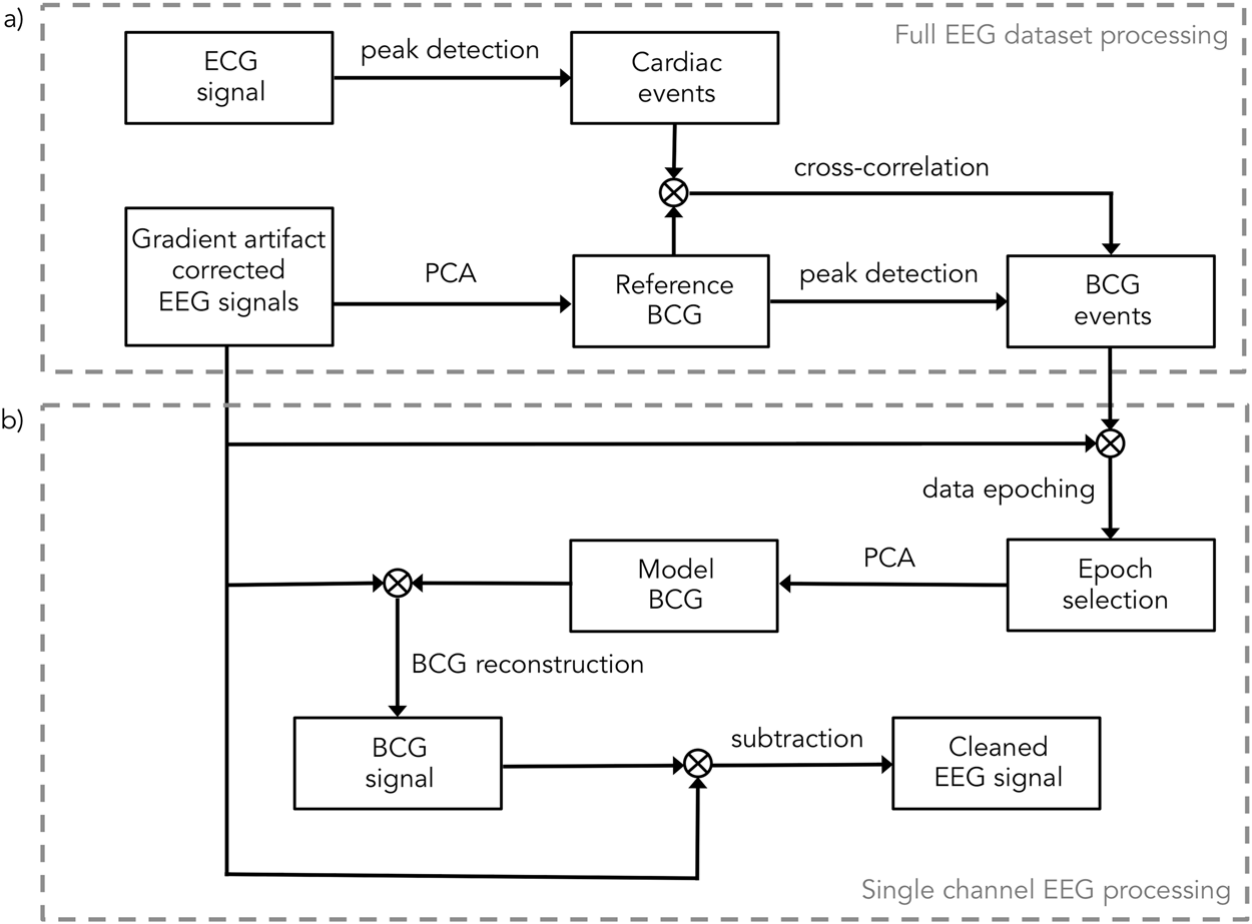
Flowchart of the method for removing the BCG artifact from the EEG data. (**a**) The ECG signal is processed to detect the cardiac events. EEG data are given as input for PCA to generate a reference BCG signal, defined as the first principal component. Information on cardiac occurrences is used to retrieve the BCG occurrences, by aligning the cardiac events detected from the ECG signal with the reference BCG. The reference BCG can be treated as an alternative ECG signal and be processed to detect the cardiac events. The detection of cardiac occurrences based on EEG information can take into account of the subject- and epoch-specific variability of the delay between cardiac events in the ECG signal and EEG data. (**b**) The subtraction of this template from gradient-corrected EEG data leads to clean EEG signals. Information on cardiac occurrences, previously obtained, is used to epoch the EEG data channel-by-channel. Epoched single EEG channel is processed to generate an epoch-by-epoch BCG artifact model. The artifact model is combined with the original data for reconstructing the full BCG artifact time-course. An artifact-corrected EEG signal is obtained by subtracting the BCG artifact time-course from the input EEG signal. The procedure in (**b**) is separately performed for each channel.

In this study, we propose an adaptive OBS (aOBS) method for BCG artifact removal, which addresses the main limitations of the OBS method. Specifically, the aOBS method attenuates the BCG artifact from EEG recordings acquired inside the MR scanner following two sequential steps: (1) *Detection of BCG occurrences* (Fig. 1a): ECG peaks are detected, and this information is subsequently used to guide the identification of BCG peaks in gradient artifact corrected EEG data; in alternative, BCG peaks can be directly extracted from the same EEG data. (2) *BCG artifact reconstruction and subtraction* (Fig. 1b): For each channel, EEG data are epoched based on BCG peaks and then PCA is applied; PCs are retained using a selection criterion based on the explained variance, which leads to the construction of a BCG artifact basis. The BCG artifact is reconstructed epoch by epoch by linear combination of the artifact basis, and is then subtracted from the EEG data. This approach ensures a more effective artifact characterization and it automatically identifies the artifactual components to be removed from the data, based on signal features. We conduct an extensive evaluation of the performance of aOBS with respect to AAS, ICA and OBS, using high-density EEG data acquired with simultaneous fMRI during a visual stimulation paradigm.

## Results

**Figure 2:**
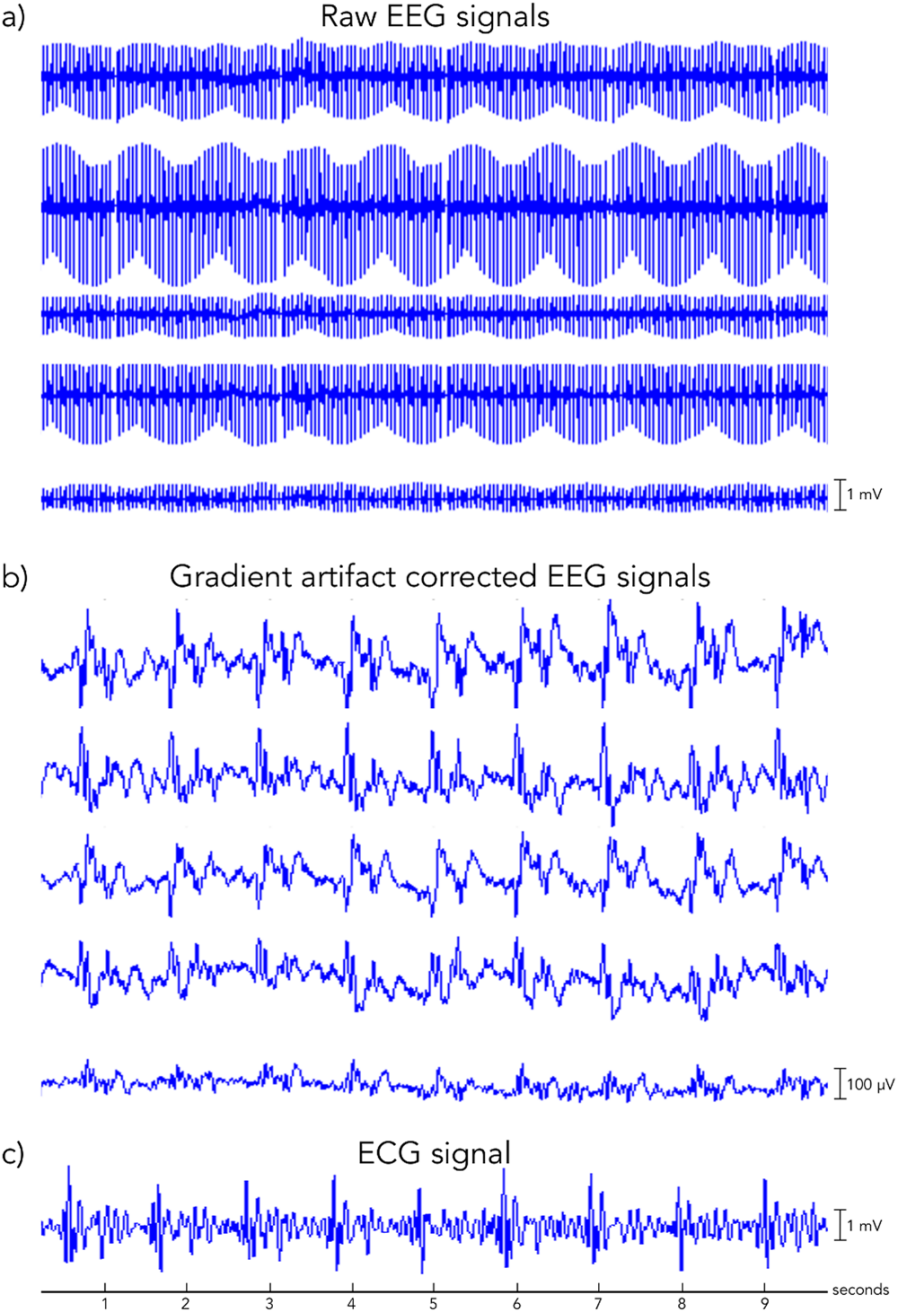
Illustrative example of 10 seconds of EEG data. Five representative channels (channels F8, F7, T4, T3, and T6, from top to bottom) are displayed: (**a**) before gradient artifact correction, i.e. raw data; and (**b**) after gradient artifact correction. In (**c**) the ECG signal concurrently recorded is displayed.

Gradient-artifact correction was successfully performed on all datasets (Fig. 2a,b). Also, the quality of artifact-corrected ECG signals was sufficiently high for the reliable detection of cardiac events (Fig. 2c). Based on these EEG and ECG data, we could therefore proceed with the assessment of aOBS, our BCG artifact removal method, against AAS, ICA and OBS.

When using aOBS, the number of components that contributed to the BCG artifact basis varied across subjects and across channels. These ranged between 1 and 6 for S1, 2 and 6 for S2, 1 and 7 for S3, 2 and 7 for S4, 3 and 8 for S5, and 1 and 6 for S6.

**Figure 3:**
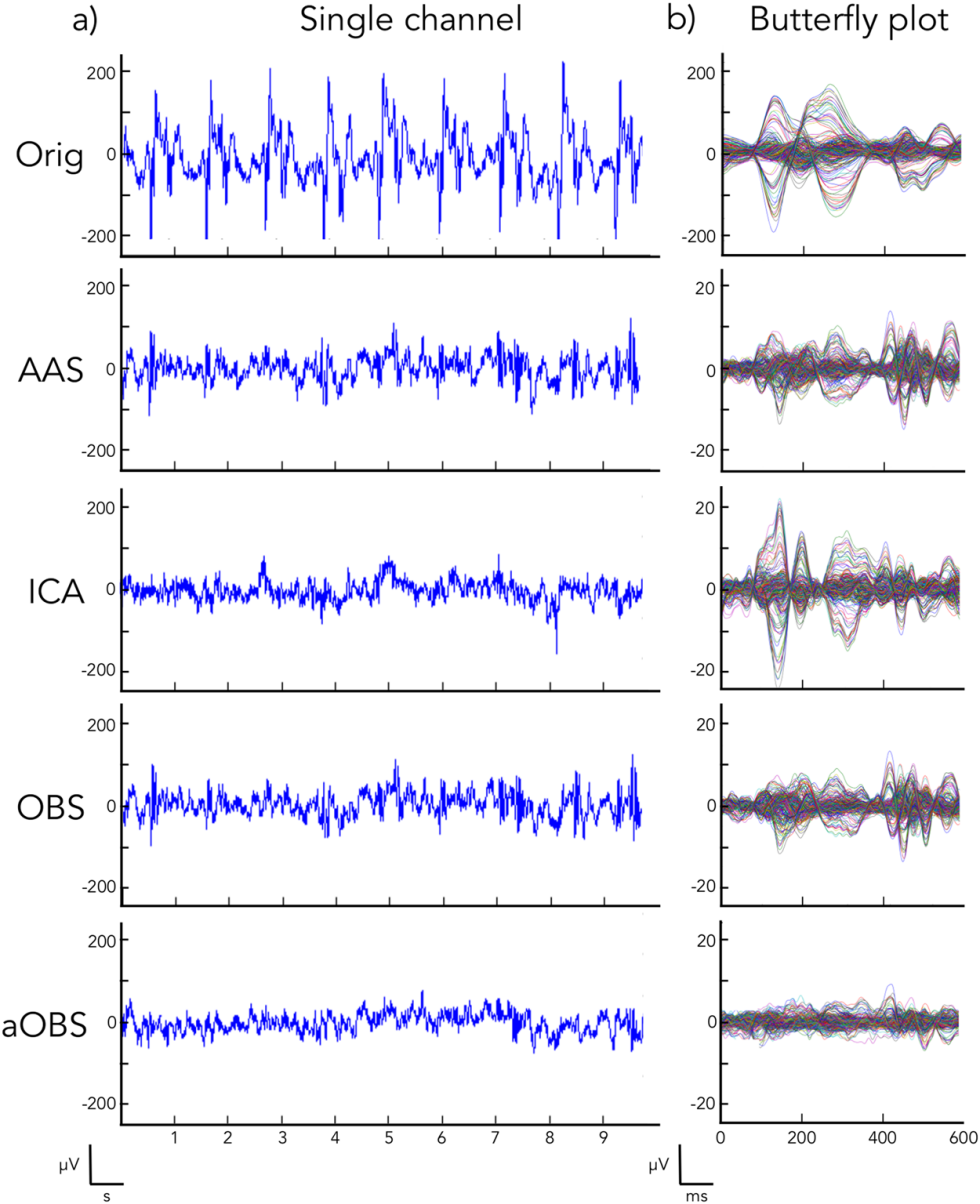
Qualitative comparison between the EEG recordings before (Orig) and after BCG artifact correction (AAS, ICA, OBS, aOBS) in a representative subject (S1). (**a**) Illustrative example of 10 s signal from a single EEG channel (channel F8). Following the application of BCG removal methods, the amplitude of the EEG signal strongly decreases, as the BCG artifact contribution is attenuated. (**b**) EEG signals from all recording channels were epoched in time-windows of 600 ms by using as triggers the cardiac events detected from the ECG signal. By displaying the cardiac evoked activity, it is possible to observe the overall cardiac contribution to the EEG data.

First, we assessed the performance of aOBS in terms of BCG artifact attenuation, by quantifying BCG residual intensity and maximum cross-correlation between EEG signals and ECG signal. Inspection of the EEG data revealed clear BCG residuals for AAS, ICA and OBS, but not for aOBS. This effect was visible both when inspecting the whole signal time-courses (Fig. 3a) and average activity time-locked to the ECG events (Fig. 3b).

**Figure 4:**
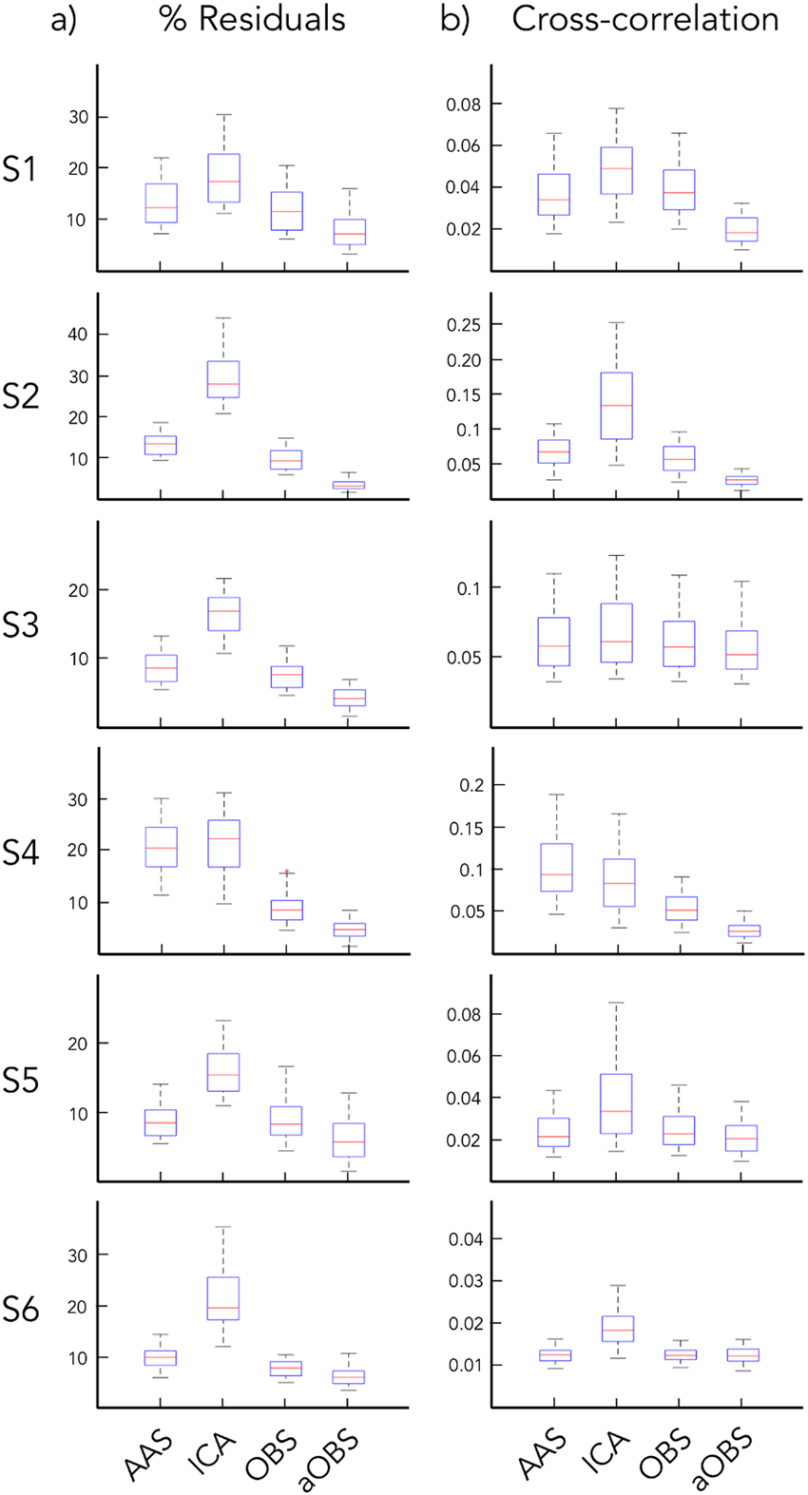
Quantitative assessment of BCG artifact attenuation for all the subjects. Box plots are provided for all the subjects (S1, S2, S3, S4, S5, S6) for which AAS, ICA, OBS, and aOBS methods were applied for removing the BCG artifact from the EEG recordings. Comparison is performed by examining: (**a**) the percentage of BCG residuals in the cleaned EEG data. For each subject, aOBS method outperforms all the other methods in terms of BCG artifact attenuation as shown by the consistently lower percentage of residuals. (**b**) the cross-correlation between EEG channels and ECG signal.

When we conducted quantitative analyses on the BCG-corrected signals, we could appreciate that BCG residuals strongly decreased when using aOBS, with average values equal to 5.53% and relatively small variability across channels for the same subject, as well as across subjects (Figs 3 and 4a). In line with qualitative analyses, other methods yielded larger BCG residuals. Specifically, AAS, ICA and OBS had an average value of 12.51%, 20.63% and 9.20%, respectively. Also the maximum value of the cross-correlation function was generally lower for aOBS than other methods (Fig. 4b). Specifically, the average value across datasets was 0.180 before BCG artifact correction; after using AAS, ICA, OBS and aOBS it decreased to 0.051, 0.067, 0.042 and 0.028, respectively.

**Figure 5:**
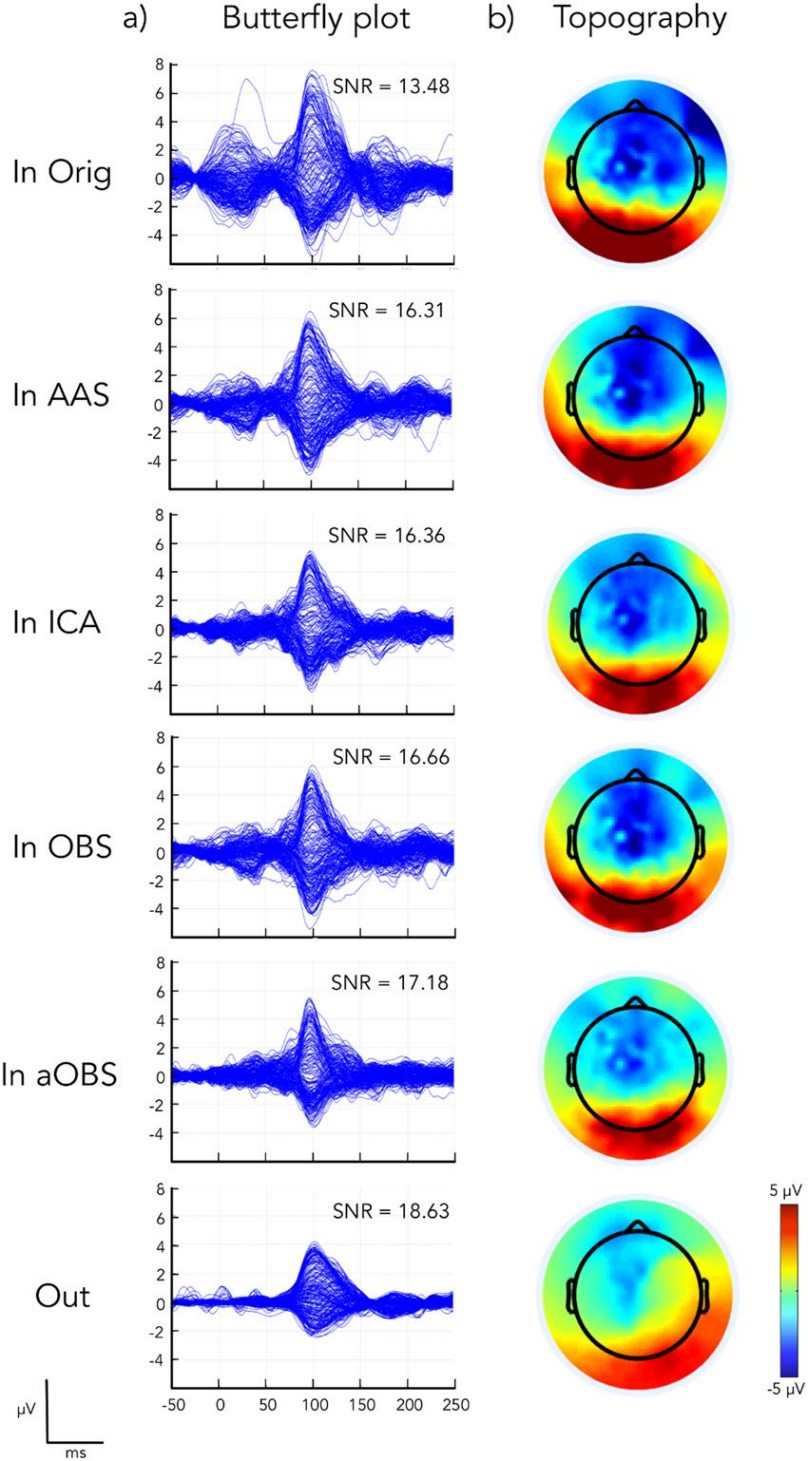
VEPs obtained from gradient-artifact corrected (Orig) and also BCG-artifact corrected (AAS, ICA, OBS and aOBS) data in a representative subject (S1). These VEP were compared with those obtained from the same subject outside the scanner (Out). EEG signals were epoched using the visual events as triggers, and baseline-corrected using the pre-stimulus interval. (**a**) Average time-courses for all recording EEG channels are presented on the left (Butterfly plot); (**b**) Topographic maps, on the right (Topography), refer to the spatial distribution across channels at the P100 latency, which commonly corresponds to the strongest visual activation.

In the assessment of aOBS performance, we also considered signal preservation as an important feature. Accordingly, we calculated and evaluated event-related potentials (ERPs) from BCG-corrected EEG data in terms of signal-to-noise ratio (SNR)^47^ and inter-trial variability^37^. A qualitative analysis revealed that both noise and signal intensities in the ERPs were generally lower for aOBS than for other methods (Fig. 5). However, the spatial topography obtained with aOBS was clearer and more in line with the activation of occipital regions following visual stimulation, which was also evident from EEG data collected from outside the scanner (Fig. 5).

**Figure 6:**
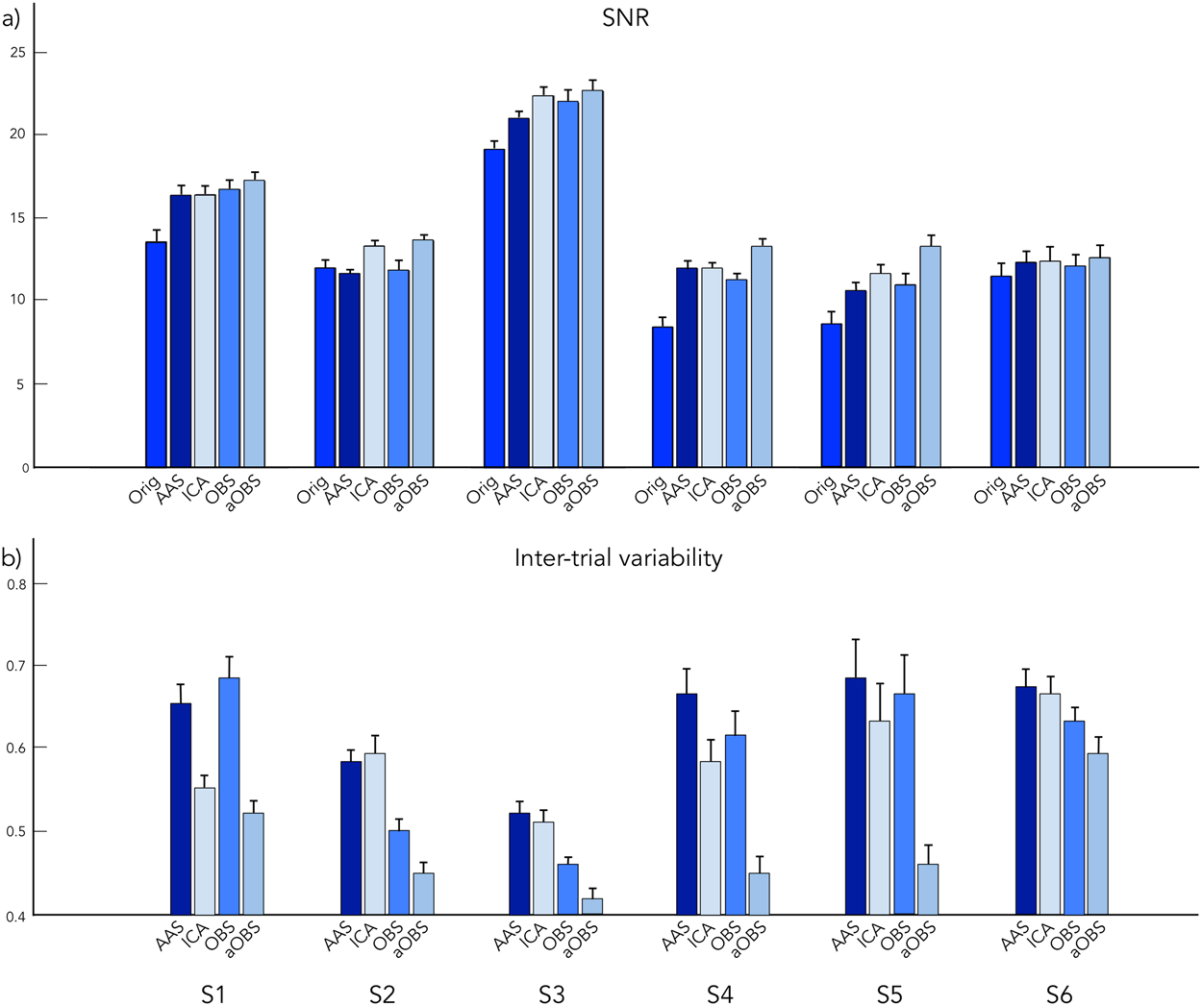
Quantitative assessment of brain signal preservation for all the subjects. (**a**) SNR (expressed in dB) and (**b**) inter-trial variability associated with the VEPs calculated from gradient-artifact corrected (Orig) and BCG artifact corrected (AAS, ICA, OBS and aOBS) data for each subject (S1, S2, S3, S4, S5, S6). Error bars indicate the standard error across occipital channels.

Furthermore, noise over frontal regions, which are strongly affected by the BCG artifact^38^, seemed to be suppressed by aOBS, and only to a lesser extent by other methods (Fig. 5b). In line with these qualitative observations, we also found that the average SNR across subjects was the largest among BCG-correction methods, leading to an improvement of 29.84%, with respect to the non-corrected data, against 16.60%, 22.18%, 17.14% for AAS, ICA and OBS, respectively (Fig. 6a). For all the subjects in the study, the inter-trial variability index of the ERPs was also lower for aOBS as compared to the other approaches (Fig. 6b), suggesting a more reliable reconstruction of task-related brain activity.

## Discussion

In this study, we presented aOBS, an enhanced method for removing the BCG artifact from EEG recordings acquired during fMRI scanning. Although some methods to address the problem of BCG contamination already exist^29,36,42^, such as AAS, ICA and OBS, none of them showed fully satisfactory results. Unlike these approaches, the aOBS method is adaptive, as it considers the presence of a varying delay between ECG and EEG signals, and it builds an artifact template separately for each channel based on the characteristics of the EEG signal. Our analyses revealed that aOBS has superior performance compared to AAS, ICA and OBS. Specifically, aOBS enables effective reduction of BCG while preserving brain signals, which enhances the signal-to-noise ratio in the EEG data collected during fMRI scanning. Since it is easy to use and it does not require parameter tuning, the aOBS method may find wide application in the field of simultaneous EEG-fMRI.

The aOBS method, similarly to OBS, builds an artifact template using a basis set created from an epoched EEG signal. However, the basis set is built in a substantially different manner as compared to OBS. First of all, BCG events are used instead of ECG events for data epoching. This permits to address the problem of a variable delay between ECG and BCG peaks^38^. In aOBS, two options are available for BCG peak detection: either a reference BCG is estimated from the EEG data and peak detection is run directly on it; alternatively, ECG peak detection is performed, and then the timing of BCG peaks is identified by using the cross-correlation function between ECG and reference BCG signal, calculated from the full EEG dataset. These approaches lead to very similar results (data not shown), but the first solution may be preferred because it does not require the availability of a high-quality ECG signal. The alignment on the EEG signal to the BCG events has a direct impact on data epoching, and therefore influences the results of PCA, where less variability across epochs generally results in a higher variance of the first PCs. Notably, PCA is run only on selected EEG epochs, excluding those with large deviation from the mean response. This choice distinguishes aOBS from OBS, in which all epochs are considered. Our solution leads to the definition of a basis set that more clearly reflects BCG activity, whereas other artefactual (but also signal) sources may be modelled by OBS. Concerning the number of PCs to be included in the basis set for OBS, it is worth noting that no consensus has been reached^37^. We found that the number of PCs included in the BCG artifact model during aOBS processing varied considerably across channels (between 1 and 8), but on average was in line with the number of PCs set in most of the studies using OBS (between 3 and 4)^37^. Interestingly, it has been shown that the selection of the same number of PCs for all the subjects fails to capture inter-individual differences. This leads to satisfactory results for some datasets, and not for others, in which non-negligible BCG residuals are still present and/or relevant neuronal activity is removed^48,49^. Furthermore, previous studies showed that EEG recordings are contaminated by the BCG artifact in a variable manner, depending on their position of the electrode on the scalp and the relative orientation of the magnetic field^34^. For this reason, the number of PCs is selected on a channel-by-channel basis in aOBS, using a threshold defined based on the Scree test^50^. Notably, this threshold is typically lower with a high number of BCG events, in line with a relatively lower probability that a PC containing brain activity has higher variance than one containing BCG activity.

The application of the aOBS method resulted in an effective removal of the BCG artifact from EEG recordings collected during fMRI scanning. Specifically, our analyses revealed reduction of the BCG artifact residual (Figs 3 and 4), accompanied by a respectable preservation of brain activity (Figs 5 and 6). Compared to commonly used BCG removal techniques, such as AAS, ICA and OBS, aOBS showed smaller BCG residuals (Figs 3 and 4a) and lower cross-correlation with ECG (Fig. 4b), as well as higher single-subject and group-level SNR (Figs 5 and 6). Similarly to previous reports^25^, we also obtained a decreased inter-trial variability in the visual evoked response. This attenuation can be directly ascribed to the attenuation of the BCG, which is not time-locked to the visual events. On the other hand, the residual inter-trial variability can be considered to be primarily of neuronal origin, and associated with spontaneous brain activity^12^. The decreased single-trial variability is of utmost importance for studies focused on the coupling between EEG and fMRI trial-by-trial variations, which have recently attracted the interest of the neuroscientific community^11,51^. Indeed, the presence of residual BCG artifacts in the EEG data is typically associated with increased single-trial variability, and with a reduced trial-by-trial correlation with fMRI-based measures.

Several previous studies compared the performance of AAS, OBS and ICA under a wide range of conditions. The results of these studies have not provided clear indications concerning which method may provide the best compromise between removal of BCG artifact and preservation of EEG physiological signal of interest. In some cases, ICA and OBS were found to perform better^36,42^ and in other cases worse than AAS^43^. Conflicting results^32^ or no significant differences^37^ were reported in the direct comparison between ICA and OBS. Also, studies examining the combination of different methods did not yield converging results^43^. We argue that this inconsistency may be mainly due to the need, for each of these methods, of setting appropriate parameters for artifact removal. In its current implementation, the aOBS method does not require parameter tuning and still enables a reliable reduction of BCG residuals while preserving brain activity. It should also be considered that aOBS can be easily combined with ICA, as well as other techniques for BCG artifact attenuation. Future studies are warranted to examine under which conditions the combination of aOBS with other methods can be effective.

Recent studies comprising high-density EEG proposed an approach for taking into account BCG spatial redundancy by modeling the BCG artifact starting from prior information in a subset of channels^52,53^. This approach is based on the assumption that the BCG at each recording channel can be reconstructed by linearly combining the BCG from other channels. However, this assumption may not be fully met, as previous literature reported that the BCG is spatially non-stationarity^30,33,38^. In fact, also in this study we revealed a large variability in the BCG artifact across subjects and, for each subject, across electrodes. Although we addressed in aOBS the problem of a varying delay between ECG and BCG events^38–40^, we did not estimate the exact delay on a channel-by-channel basis. Rather, we identified the BCG peaks using information from all EEG signals together. A channel-by-channel peak detection could not be performed as the BCG artifact shows an extremely variegated contribution depending on the scalp region in which EEG sensor is located, ranging from very well defined to barely visible peaks. Another important limitation of our study concerns the validation. It should indeed be noted that we conducted extensive investigations of several method performance using datasets collected in young healthy subjects with a 256-channel EEG system. Whereas the comparative results across BCG attenuation methods are certainly valid, it remains an unanswered question whether and to what extent the current findings in terms of BCG suppression and brain signal preservation can generalize to a larger number of datasets and to different EEG systems. Future studies are warranted to address this question. In the recent years, a growing number of studies has been dedicated to the development of hardware enhanced algorithms, including camera tracking system^49,54^, carbon wire loops^55^, and insulating reference layers^56^, which enable high-quality BCG corrections, also in real-time. Despite the clear benefits related to these approaches, the introduction of additional material inside the MR environment may increase the chance of technical issues and affect the comfort of the subject, a part from also limiting their widespread use due to the dedicated hardware required. In this sense, an effective post-processing technique, such as the aOBS, might prevent technical issues during data acquisition, while potentially providing comparable data quality as the hardware-based methods. Though, this scenario should be further explored and comparisons performed, eventually, following the implementation of aOBS also for on-line applications.

## Conclusion

In simultaneous EEG-fMRI, the presence of BCG residuals in EEG data represents a serious problem. In fact, a commonly used analysis approach for EEG-fMRI data integration consists of extracting EEG power fluctuations, convolving them with a standard hemodynamic response and correlating them with fMRI time-courses^12,57^. In this case, the presence of BCG residuals, which introduce biases in the power spectrum of the EEG data, might potentially lead to misleading interpretation of the EEG-fMRI relationship. To address this problem, we developed aOBS, an enhanced method for BCG artifact attenuation. While conceiving such a method, we specifically considered the temporal and spatial features of the BCG^38^, and implemented adaptive solutions for BCG artifact detection and selection of an optimal basis set. As compared to alternative methods, the application of aOBS led to a remarkable BCG artifact attenuation and a substantial preservation of brain signal. Given its performance in terms of BCG attenuation, we suggest that aOBS may find wide application in simultaneous EEG-fMRI studies. In particular, its use might be fundamental in resting state EEG-fMRI studies, in which no experimental events are present and no signal averaging is therefore possible.

## Methods

### Method description

The aOBS method attenuates the BCG artifact from EEG recordings acquired inside the MR scanner following two sequential steps: *Detection of BCG occurrences* (Fig. 1a) and *BCG artifact reconstruction and subtraction* (Fig. 1b). The full procedure is explained in detail in the following paragraphs.

#### Detection of BCG occurrences

The aOBS method requires the definition of BCG peak timing, to take into account variable time delays between ECG peaks and BCG events. We therefore implemented a method for robustly detecting BCG events. Two different options are available, the first of which uses both ECG and EEG signals, whereas the second relies on the EEG signals only. Following the first option, the ECG signal is filtered using a Finite Impulse Response (FIR) filter in the band between 5 and 20 Hz. Then, the power of the signal, estimated as the square of the signal amplitude for each time sample, is calculated and band-pass filtered between 0.1 and 2 Hz. ECG peaks are defined as local maxima in the band-pass filtered power time-series. This enables to reduce the complexity of the data and, thus, enhance the chance of correct peak detection. False positives (less than 0.2% of the total) and negatives (less than 0.2% of the total) are detected and eliminated by using information on the heart rate variability of the individual dataset. Specifically, an upper limit for each cardiac interval is set to 1.5 times the median of all the cardiac intervals. When a cardiac interval exceeds this duration, one of more peaks are added, such that the duration of each new cardiac interval is equal and in the normal range. Also, a lower limit for each cardiac interval is set to 0.6 times the median of all the cardiac intervals. When a cardiac interval is below this threshold, the second peak defining the cardiac interval is eliminated. Next, the average EEG signal triggered to the ECG peaks is calculated and used to detect the BCG peaks by defining the latency of maximum cross-correlation with each EEG epoch. This allows compensating the effect of positive and negative offsets that may rise from the detection of the peaks from the power of the signal and ensuring exact peaks alignment. When using the second option for BCG peak detection, PCA decomposition is run on the whole EEG dataset, and the procedure described for processing the ECG signal is applied on the timecourse of the first PC. The BCG is always very prominent, such that the same peak detection procedure described above can be used to directly obtain the BCG event timing.

#### BCG artifact reconstruction and subtraction

The signal from a single EEG channel is epoched using the BCG events as triggers, with the epoch length corresponding to the maximum duration between consecutive BCG events for each participant. This choice is due to the need of modeling the BCG artifact also for the longest epochs, such that no discontinuities are present when the BCG signal is reconstructed. Following this initial step, epoched EEG data are correlated to their average, such that good and bad epochs (e.g. epochs contaminated by eye blinks) can be separated using the Tukey method^58^. PCA is run on the good trials only, and the PCs to be retained are defined on the basis of the Scree test^50^. The retained PCs are stacked together to form a basis set, which is subsequently used to calculate weights for each epoch using linear regression. These weights are then used in combination with the basis set to reconstruct the BCG artifact time-course. The latter is finally subtracted from the BCG-corrupted EEG signal to obtain a clean EEG signal.

### Method validation

#### Subjects and experimental paradigm

Six right-handed healthy volunteers (age 25.5 ± 1.64 years, 3 males and 3 females) participated in the experiment. All of them reported normal or corrected-to-normal vision, and had no psychiatric or neurological history. Before undergoing the examination, they gave their written informed consent to the experimental procedures, which were approved by the Medical Ethics Committee of UZ Leuven. During the experiment, which was conducted in accordance with the relevant guidelines and regulations, the volunteers were presented with visual stimuli (30 s), alternated with periods of rest (30 s), for 6 minutes in total. Visual stimulation was performed by a squared full contrast flashing checkerboard (2 Hz reversing frequency) projected on a translucent screen, which was visible by the volunteer through a mirror. The visual stimuli had 7°-wide field of view, and were prepared using the Psychtoolbox 3 (http://psychtoolbox.org/). A white fixation cross was kept on the center of the screen to minimize eye movements. The exact timing of the stimulus presentation was extracted by using a photoelectric cell.

#### EEG-fMRI data acquisition

fMRI data acquisition was performed a 3 T Philips Achieva MR scanner (Philips Medical Systems, Best, the Netherlands)) using a T2*-weighted SENSE sequence. The scanning parameters were TR = 2000 ms, TE = 30 ms, 36 slices, 80 × 80 matrix, voxel size 2.75 × 2.75 × 3.75 mm^3^, flip angle = 90 degrees. During the recording, the helium pump of the magnet was switched off for the full duration of the functional acquisition. EEG signals were recorded by the MR-compatible 256-channel HydroCel Geodesic Sensor Net (GSN) (EGI, Eugene, Oregon, USA). The impedance of each electrode was maintained lower than 50 kΩ across the full recording by soaking the sponge contained in each electrode with a saline solution. The contact of the EEG electrodes with the patient scalp was maintained by placing an elastic bandage above the EEG net. The electrocardiographic (ECG) signal was also acquired with two MR-compatible electrodes positioned on the chest, in correspondence to the apical and the left side of the heart, respectively. The EEG and ECG cables were connected to the EEG amplifier, which was contained in a field isolation containment system (FICS) and positioned next to the MR bore. EEG data were collected at a sampling rate of 1 KHz, and were synchronized to the MR scanner internal clock. The acquired signals were sent via an optical cable to the EEG recording computer outside the MR scanner room.

#### EEG data preprocessing

Processing of the EEG data was carried out using the EEGLAB toolbox (https://sccn.ucsd.edu/eeglab/)^59^, as well as built-in functions of MATLAB (MathWorks, Natick, US). First of all, gradient artifact attenuation was performed by using the fMRI artifact slice template removal (FASTR) method implemented in EEGLAB^36^. Subsequently, EEG data were digitally filtered in the band [1–70 Hz] and re-referenced to average reference^60^. At this stage, different methods were applied for the attenuation of the BCG artifacts. In addition to the aOBS method, we processed the EEG data using standard implementations of commonly used BCG artifact removal methods, including AAS^29^, OBS^36^, and ICA^42^. For AAS and OBS, we used the FMRIB plug-in of EEGLAB^36^. AAS was performed by considering a number of windows equal to 20^43^. OBS was run by setting the number of retained PCs equal to 3^34^. ICA-based artifact removal was performed using the FastICA algorithm^61^. The ICs showing a maximum cross-correlation with the ECG signal larger than 0.2 were regressed out of the EEG signals^47^.

#### Assessment of method performance

We tested the performance of aOBS, AAS, ICA and OBS in terms of BCG artifact attenuation. To this end, we considered two different metrics: (1) maximum cross-correlation between EEG signals and ECG signal, and (2) BCG residual intensity. The first metric was calculated using a procedure similar to the one for selecting the BCG-related ICs previously described. BCG residual intensity was quantified by calculating the root mean square (RMS) of the EEG data, epoched over windows of 600 ms after the ECG peaks and averaged^32,42^. The percentage of BCG residual intensity was defined as the ratio between RMS of EEG data after and before BCG correction, respectively.

We also assessed neural signal preservation, as stronger reduction of the BCG artifact may be associated with an overall signal reduction, including brain signal. To this end, we reconstructed the evoked response associated with the visual stimulation. We focused our analyses on occipital channels, where evoked neural activity was expected to be most prominent. We epoched the EEG data over windows of 400 ms starting 100 ms before each visual event. For each occipital channel^60^, we calculated the signal-to-noise ratio (SNR)^47^, defined as the ratio between the maximum RMS in the evoked response period (latency between 80 and 120 ms), and the average RMS in the pre-stimulus interval (from −100 to 0 ms). Since BCG residuals are randomly spread over trials^36^, the better the artifact is removed, the smaller the single-trial variation should be. Accordingly, we also calculated an inter-trial variability index, defined as the ratio between the across-trial standard deviation after and before BCG removal, respectively^37^.

### Data availability

The datasets generated during and/or analysed during the current study are available from the corresponding author on reasonable request.
